# Relationship between Hypothyroidism and Non-Alcoholic Fatty Liver Disease: A Systematic Review and Meta-analysis

**DOI:** 10.3389/fendo.2017.00335

**Published:** 2017-11-29

**Authors:** Weiwei He, Xiaofei An, Ling Li, Xiaoqing Shao, Qian Li, Qiuming Yao, Jin-an Zhang

**Affiliations:** ^1^Department of Endocrinology, Affiliated Hospital of Yanan University, Shanxi, China; ^2^Department of Endocrinology, Jinshan Hospital of Fudan University, Shanghai, China; ^3^Department of Endocrinology, Shanghai University of Medicine & Health Sciences Affiliated Zhoupu Hospital, Shanghai, China

**Keywords:** hypothyroidism, subclinical hypothyroidism, non-alcoholic fatty liver disease, meta-analysis, relationship

## Abstract

**Background:**

Previous studies propose that hypothyroidism might play a crucial role in the pathogenesis of non-alcoholic fatty liver disease (NAFLD), but findings from published studies on the relationship between hypothyroidism and NAFLD are still controversial. Our study aimed to make a comprehensive evaluation of the relationship between hypothyroidism and NAFLD through a meta-analysis.

**Methods:**

PubMed, China Dissertation Database, and EMBASE databases were searched to find observational studies assessing the relationship between hypothyroidism and NAFLD. The pooled odds ratios (ORs) with 95% confidence intervals (95% CIs) were calculated to evaluate the strength of the relationship between hypothyroidism and NAFLD through meta-analysis.

**Results:**

Thirteen articles were ultimately included in our meta-analysis. Meta-analysis of the 13 studies found a high correlation between hypothyroidism and NAFLD (OR = 1.52, 95% CI 1.24–1.87, *P* < 0.001). Meta-analysis of 9 studies providing adjusted ORs found that hypothyroidism was independently correlated with NAFLD (OR = 1.72, 95% CI 1.32–2.23, *P* < 0.001). Subgroup analysis found that both overt hypothyroidism and subclinical hypothyroidism were significantly correlated with NAFLD, and the pooled ORs were 1.70 (95% CI 1.23–2.36, *P* = 0.002) and 1.40 (95% CI 1.10–1.77, *P* = 0.006), respectively. Besides, meta-analysis of studies providing adjusted ORs also found that both overt hypothyroidism and subclinical hypothyroidism were independently correlated with NAFLD, and the pooled ORs were 1.81 (95% CI 1.30–2.52, *P* < 0.001) and 1.63 (95% CI 1.19–2.24, *P* = 0.002), respectively.

**Conclusion:**

The meta-analysis provides strong epidemiological evidence for the relationship between hypothyroidism and NAFLD. Both individuals with subclinical and overt hypothyroidism are at higher risk for NAFLD than euthyroid subjects.

## Introduction

The prevalence of non-alcoholic fatty liver disease (NAFLD) has increased substantially during the past decades, and it has become the leading cause of liver disease worldwide, which may be partly attributed to the rising prevalence of obesity (1). NAFLD is a chronic liver disease defined as hepatic accumulation of fat in the absence of excess alcohol consumption and not only insulin resistance (IR) but also genetic predisposition play a key role in its pathogenesis (1). NAFLD can be divided into two main histological categories, namely nonalcoholic fatty liver and nonalcoholic steatohepatitis, which is the progressive subtype of NAFLD and can further induce liver cirrhosis and hepatocellular carcinoma (2). An increasing number of diseases have been reported to be linked to NAFLD, such as cardiovascular disease, type 2 diabetes, chronic kidney disease, and cancer (3–5). The prevention and treatment of NAFLD have become the focus of medical research in recent years, and identifying the risk factors for NAFLD is critical to develop effective preventive interventions against NAFLD.

Hypothyroidism is a common disease of the endocrine system that affects lifelong health. The physiological role of the thyroid gland has been taken seriously by many scholars, not just because of the critical role of thyroid hormones in cell metabolism and energy homeostasis (6) but also for the more important fact that thyroid dysfunction is associated with numerous diseases (7). Hypothyroidism comprises subclinical hypothyroidism and overt hypothyroidism. Subclinical hypothyroidism is considered as a disease with an elevated thyroid-stimulating hormone (TSH) level than normal range, normal serum free thyroxine (fT4) level and absence of obvious clinical manifestation. Overt hypothyroidism is defined as a disease with an elevated TSH level and a lower fT4 level, and it may be accompanied by obvious clinical symptoms. Some studies have found that both overt hypothyroidism and subclinical hypothyroidism are associated with cardiovascular diseases and mortality. Other studies also have found that either overt hypothyroidism or subclinical hypothyroidism may be associated with other diseases, such as chronic kidney disease, dementia, and fractures (8–10).

Previous studies propose that hypothyroidism might play a crucial role in the pathogenesis of NAFLD. Some studies report that the prevalence of hypothyroidism is from 15.2 to 36.3% among patients with NAFLD, indicating that hypothyroidism is a common concomitant disease of NAFLD and may be related to the development of NAFLD (11). At present, there are a number of observational studies which have explored the relationship between hypothyroidism and NAFLD. Some studies suggested a strong correlation between hypothyroidism and NAFLD (12, 13), but there were also studies pointing out that there was no correlation (14, 15). Therefore, the association between hypothyroidism and NAFLD risk remains in dispute up to now. Thus, it is necessary to make certain about the relation between hypothyroidism and NAFLD risk through a meta-analysis.

## Materials and Methods

### Search Strategy

We followed the PRISMA method for conducting a meta-analysis of observational studies (16). In order to select the appropriate studies to be included in our meta-analysis, we searched for published studies in PubMed, China Dissertation Database, and EMBASE databases using the following keywords and terms: (hypothyroidism or thyroid dysfunction or thyroid stimulating hormone or TSH) and (nonalcoholic fatty liver disease or non-alcoholic fatty liver disease or NAFLD or fatty liver). All studies we had searched were published before May 2017, and there was no imposed restriction on country or ethnicity.

### Inclusion and Exclusion Criteria

Our inclusion criteria were as follows: (1) cohort, cross-sectional, or case–control studies which investigated the association between hypothyroidism and NAFLD; (2) all studies must report odds ratios (ORs) with 95% confidence intervals (95% CIs) values or other values which could be converted into ORs; (3) included NAFLD patients must be diagnosed with an ultrasound examination or pathologic examination to make a clear definite diagnosis, and other diseases that could cause hepatic steatosis were excluded; and (4) the diagnosis of hypothyroidism must be based on biochemical tests including TSH levels and T4/FT4 levels.

### Data Collection and Quality Assessment

The final data were abstracted from each study using standardized form: the first author’s name, year of publication, study design, study location, number of participants, participant baseline characteristics (age and gender), method used to identify and verify NAFLD as well as thyroid function, definition of hypothyroidism including overt hypothyroidism or subclinical hypothyroidism. We used Newcastle Ottawa Scale to evaluate the quality of included studies (17). The selection of studied individuals, the comparability between exposed and non-exposed individuals, and the assessment of outcomes were evaluated (17). Studies scoring 7–9 points were considered to have high quality.

### Statistical Analysis

We used ORs with 95% CIs to test the relationship between hypothyroidism and NAFLD. Study-specific ORs were pooled using meta-analysis. The heterogeneity was evaluated by *I*^2^ statistic and Cochran’s *Q*-test, and *I*^2^ more than 50% or *P* of *Q*-test less than 0.10 showed significant heterogeneity (18, 19). DerSimonian-Laird random-effect meta-analysis was adopted when obvious heterogeneity existed (20). Mantel-Haenszel fixed meta-analysis was adopted when no obvious heterogeneity existed (21). For the existence of obvious heterogeneity, subgroup analyses and meta-regression analyses were performed to explore possible sources of heterogeneity. We conducted subgroup analyses for overt hypothyroidism and subclinical hypothyroidism, respectively. In the meta-regression analyses, study design, country, number of participants, adjusted estimates and types of hypothyroidism were used as covariates. Sensitivity analysis was implemented by excluding those studies with low scores of quality. Publication bias was evaluated by funnel plot and Egger’s test (22). STATA 12.0 (StataCorp, College Station, TX, USA) was utilized in data analysis, and *P* < 0.05 was considered statistically significant.

## Results

### Study Selection

**Figure 1:**
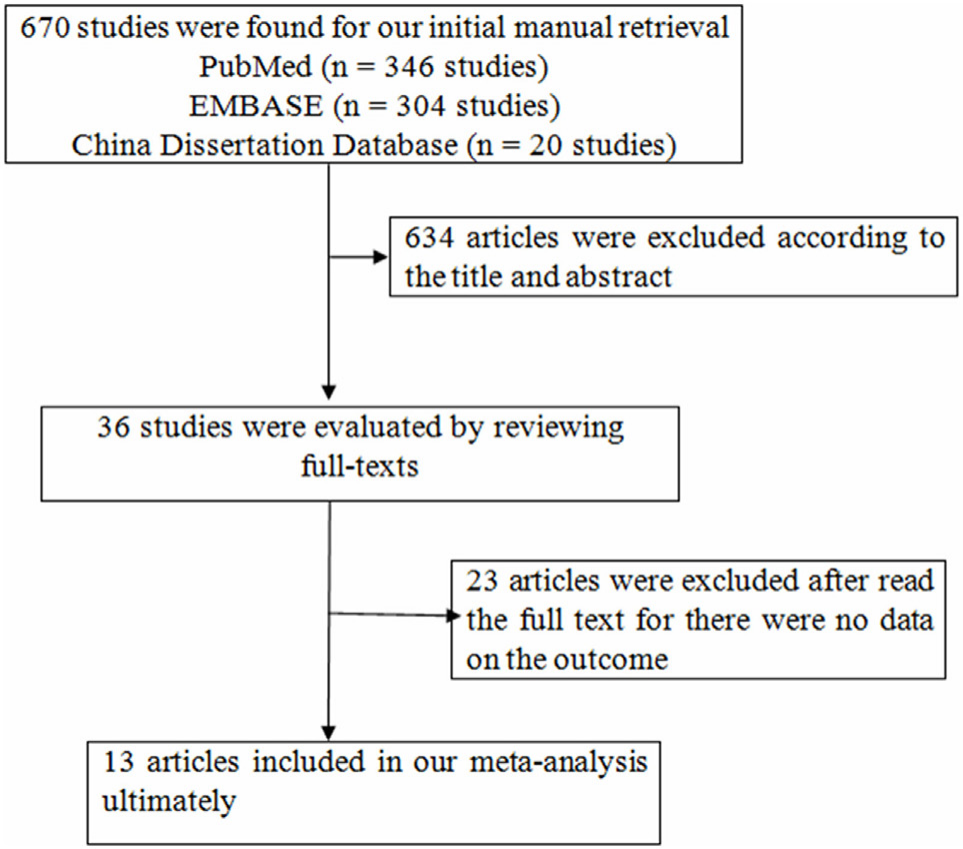
Flowchart of study selection in the meta-analysis.

We searched the databases from mentioned above and found 670 articles, while 634 articles were excluded according to the title and abstract for the following reasons: animal studies, the main purpose of these studies unrelated to the content of the present study (Figure 1). Thirty-six studies were evaluated by reviewing full-texts. Twenty-three articles were excluded after reading full-texts, for there were no data on the outcomes of interest (11, 15, 23–43). There were 13 articles included in our meta-analysis ultimately (12–14, 44–53). Following the aforementioned search, a total of 13 studies with 42,143 participants were incorporated into our final analysis studies.

### Study Characteristics

Table 1 shows the characteristics of 13 studies on the association between hypothyroidism and NAFLD (Table 1). Nine studies reported outcomes on the association between subclinical hypothyroidism and NAFLD, and six studies reported outcomes on the association between overt hypothyroidism and NAFLD (Table 1). Those 13 studies were published from 2003 to 2017, and the number of recruited participants was from 332 to 18,544. Nine studies provided adjusted ORs and four studies only provided native ORs. The quality of the included studies was shown in Table 1, and nine studies had high quality (Table 1).

**Table 1:** Characteristics of studies on the association between hypothyroidism and NAFLD.

Reference	Country	Study design	Study sample (mean age; female, %)	Diagnosis of NAFLD	Definition of hypothyroidism	Adjusted factors	Quality
Bano et al. (52)	Netherlands	Cohort	9,419 individuals (64.7 years; 56.5%)	Ultrasound	Subclinical hypothyroidism was defined as serum TSH > 4.0 mIU/L and FT4 levels within the reference range. Overt hypothyroidism was defined as serum TSH > 4.0 mIU/L and FT4 levels < 0.85 ng/dl	Age, sex, cohort, follow-up time, use of hypolipidemic drugs and cardiovascular	9

Chung et al. (12)	Korea	Cross-sectional	4,648 individuals (48.6 ± 11.8; 62.4%)	Ultrasound	Subclinical hypothyroidism (TSH > 4.1 mIU/L; normal free T4 concentration); Overt hypothyroidism: fT4 level < 0.7 ng/dL	Some known risk factors of NAFLD	8

Lee et al. (44)	Korea	Cohort	18,544 individuals (37.8 ± 5.7; 50%)	Ultrasound	Subclinical hypothyroidism (TSH > 4.2 mIU/L, normal fT4); overt hypothyroidism (TSH > 4.2 mIU/L, fT4 < 10.97 ng/dL)	Sex, age, BMI, TGs, and HDL	9

Pacifico et al. (45)	Italy	Cross-sectional	402 individuals (6–16 years)	Ultrasound	Subclinical hypothyroidism (TSH > 4.1 mIU/L with normal FT4); overt hypothyroidism (TSH > 4.1 mIU/L with FT4 < 0.7 ng/dL)	Age, gender, pubertal status, and BMI-SDS (or WC) as well as FT3 and FT4	7

Kaltenbach et al. (46)	Germany	Cross-sectional	332 individuals including 99 NAFLD patients (14.1 ± 1.9; 33.3%) and 233 non-NAFLD subjects (13.9 ± 1.8; 58.8%)	Ultrasound	Subclinical hypothyroidism (TSH > 4 μU/mL, normal thyroxine)	Age, BMI-SDS, and stage of puberty	7

Liangpunsakul and Chalasani (47)	US	Case–control	616 individuals (49 ± 13; 59%)	Enzymatic procedures	Overt hypothyroidism	Diabetes mellitus, hyperlipidemia, and hypertension	7

Pagadala et al. (13)	US	Case–control	663 individuals (50.4; 56.2%)	Histological	Overt hypothyroidism	Gender, ethnicity, diabetes, HTN, hyperlipidemia, and hypothyoidism and mean (SD)	7

Parikh et al. (48)	Western India	Case–control	800 individuals including 500 NAFLD patients (44.3 ± 3.2; 64.6%) and 300 controls (41.6 ± 3.89; 66%)	Ultrasound	Subclinical hypothyroidism (TSH > 5.5 IU/mL but <10 IU/mL) and overt hypothyroidism (TSH > 10 IU/mL)	Age, gender, alcohol use, and serum triglycerides	7

Xu et al. (49)	China	Case–control	654 individuals including 327 subclinical hypothyroidism patients and 327 controls	Ultrasound	Subclinical hypothyroidism (TSH > 4.5 mIU/L; normal thyroxine level)	Waist circumference, systolic blood pressure, diastolic blood pressure, triglyceride, HDL cholesterol, and fasting plasma glucose	7

Posadas-Romero et al. (50)	Mexico	Cross-sectional	753 individuals including 133 NAFLD cases and 620 controls (51.9; 63.9%)	Enzymatic procedures	Subclinical hypothyroidism (TSH > 4.5 mIU/L; normal thyroxine level)	None	6

Ittermann et al. (51)	Germany	Cross-sectional	3,661 individuals	Ultrasound	Hypothyroidism was defined by increased serum TSH concentrations and decreased FT3 or FT4 concentrations	None	6

Eshraghian et al. (14)	Iran	Cross-sectional	832 individuals including 127 NAFLD patients (48.2 ± 12.8) and 705 controls (36.9 ± 18.7) (61.3%)	Ultrasound	Subclinical hypothyroidism (TSH > 5.2 mIU/L, normal fT4); overt hypothyroidism (TSH > 5.2 mIU/L, fT4 < 11.5 ng/dL)	None	6

Wang and Zhao (53)	China	Cross-sectional	806 individuals (56.99 ± 7.98; 81.3%)	Ultrasound	Subclinical hypothyroidism (TSH > 4.2 μUmL, FT4: 12–22)	None	6

### Meta-Analysis

**Figure 2:**
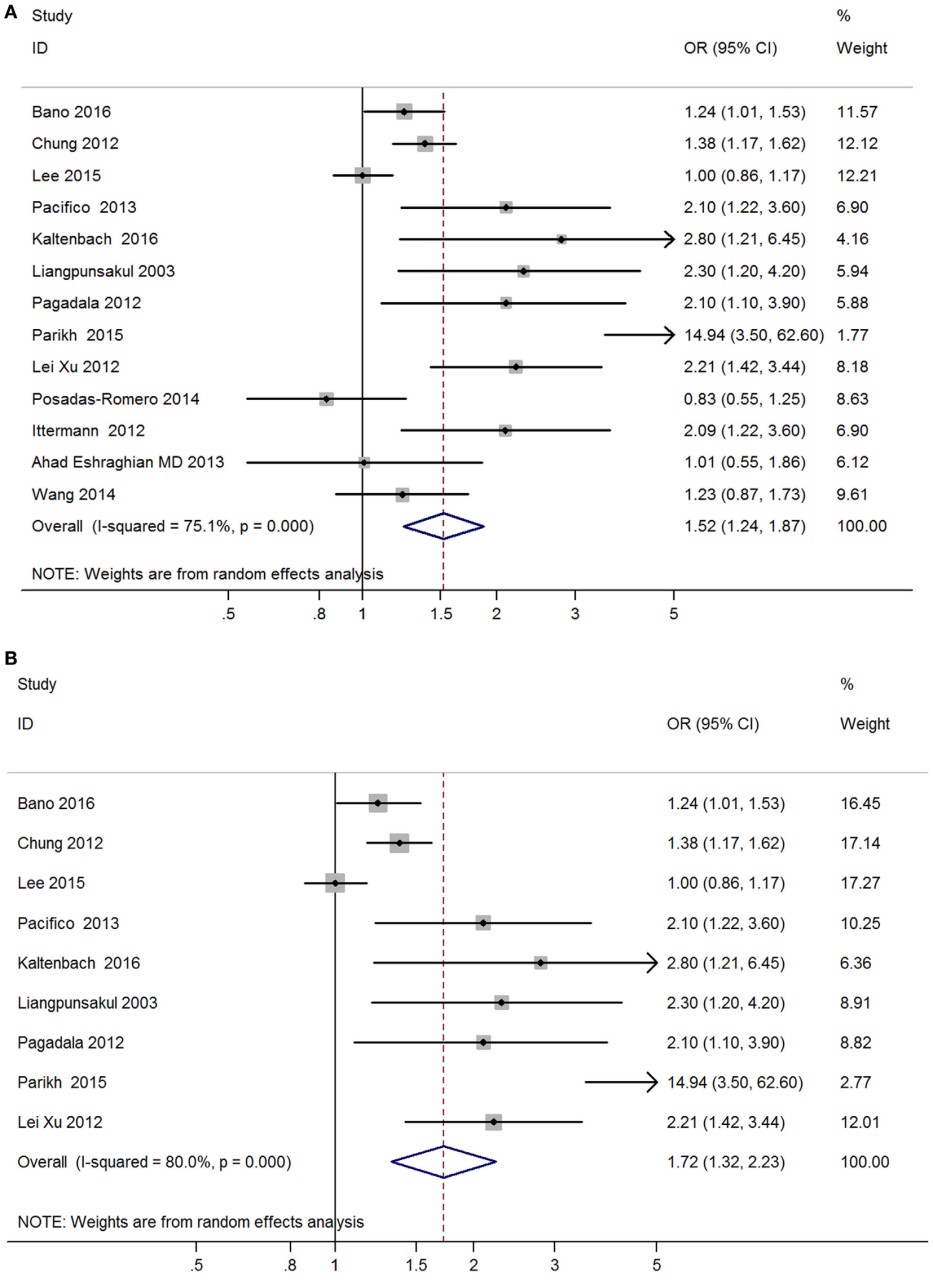
Forest plots in the meta-analysis of the relationship between hypothyroidism and non-alcoholic fatty liver disease (NAFLD). **(A)** Forest plot suggested that hypothyroidism was correlated with NAFLD. **(B)** Forest plot suggested that hypothyroidism was independently correlated with NAFLD.

Meta-analysis of the 13 studies found a high correlation between hypothyroidism (including both overt hypothyroidism and subclinical hypothyroidism) and NAFLD (OR = 1.52, 95% CI 1.24–1.87, *P* < 0.001) (Figure 2A, Table 2). After excluding 4 studies without adjusted ORs, meta-analysis of 9 left studies found that hypothyroidism was significantly and independently correlated with NAFLD (OR = 1.72, 95% CI 1.32–2.23, *P* < 0.001) (Figure 2B).

**Table 2:** Summary of the main findings in the meta-analysis of the association between hypothyroidism and NAFLD.

Outcomes	Studies (participants)	Pooled estimates		Heterogeneity	
		OR (95% CI)	*P*-value	*I*^2^	*P*
**Hypothyroidism**					
Total studies	13 (42,143)	1.52 (1.24–1.87)	<0.001	75.1%	<0.001
Studies with adjustment	9 (36,078)	1.72 (1.32–2.23)	<0.001	80.0%	<0.001
**Overt hypothyroidism**
Total studies	6 (34,735)	1.70 (1.23–2.36)	<0.002	37.9%	0.153
Studies with adjustment	5 (33,903)	1.81 (1.30–2.52)	<0.001	36.3%	0.179
**Subclinical hypothyroidism**					
Total studies	9 (36,390)	1.40 (1.10–1.77)	<0.006	73%	<0.001
Studies with adjustment	6 (33,999)	1.63 (1.19–2.24)	<0.002	80.6%	<0.001

Obvious heterogeneity was found among these 13 studies (*I*^2^ = 75.1%; *P* < 0.001, Table 2). In the meta-regression analyses, we found that study design was a possible source of heterogeneity (*P* = 0.16), but other covariates were not.

**Figure 3:**
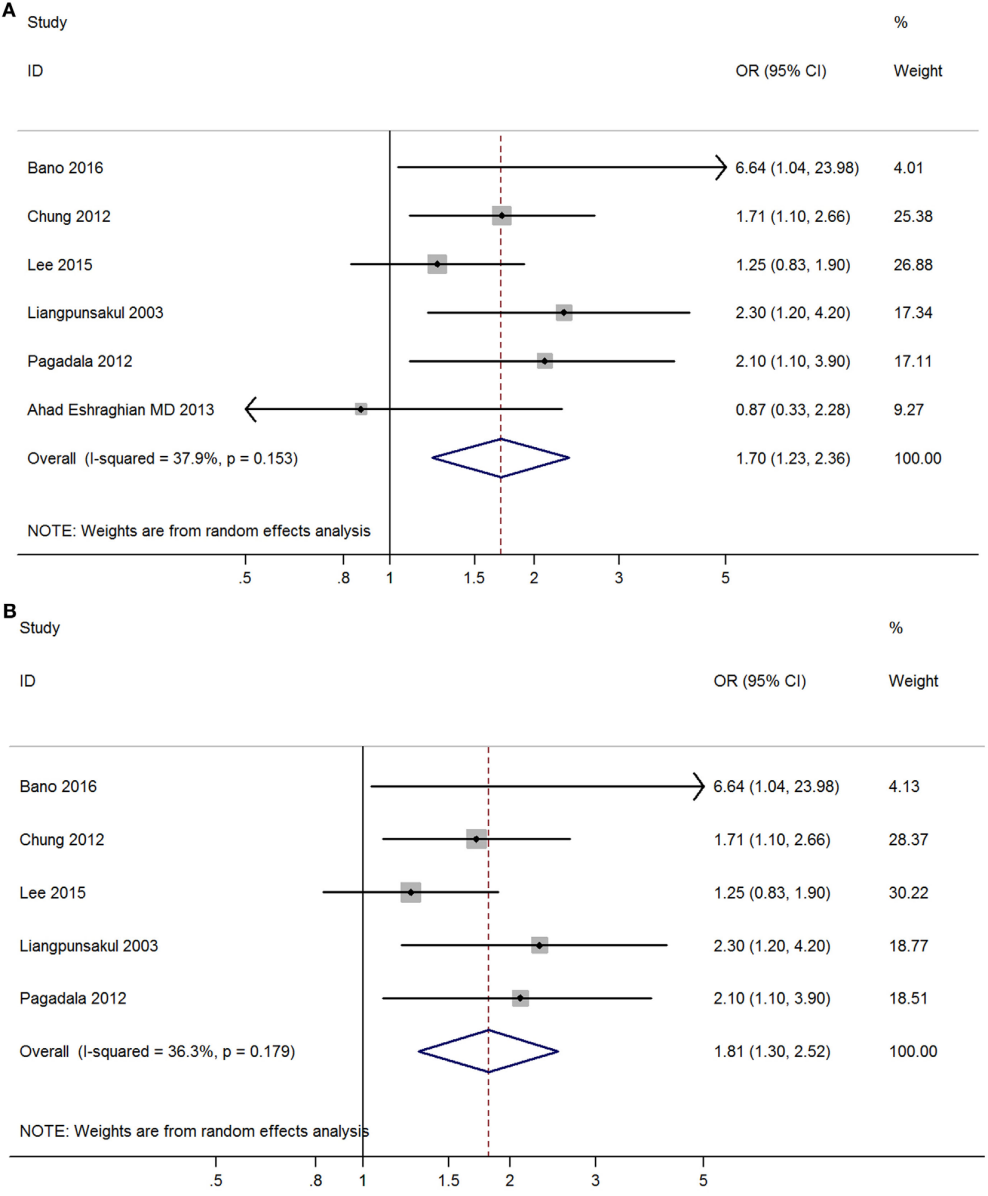
Forest plots in the meta-analysis of the relationship between overt hypothyroidism and non-alcoholic fatty liver disease (NAFLD). **(A)** Forest plot suggested that overt hypothyroidism was correlated with NAFLD. **(B)** Forest plot suggested that overt hypothyroidism was independently correlated with NAFLD.

Meta-analysis of six studies found that overt hypothyroidism was significantly correlated with NAFLD (OR = 1.70, 95% CI 1.23–2.36, *P* < 0.002) (Figure 3A, Table 2). After excluding one study without adjusted ORs, meta-analysis of the other five studies found that overt hypothyroidism was significantly and independently correlated with NAFLD (OR = 1.81, 95% CI 1.30–2.52, *P* < 0.001) (Figure 3B). No obvious heterogeneity was found among those five studies (*I*^2^ = 36.3%; *P* = 0.179).

**Figure 4:**
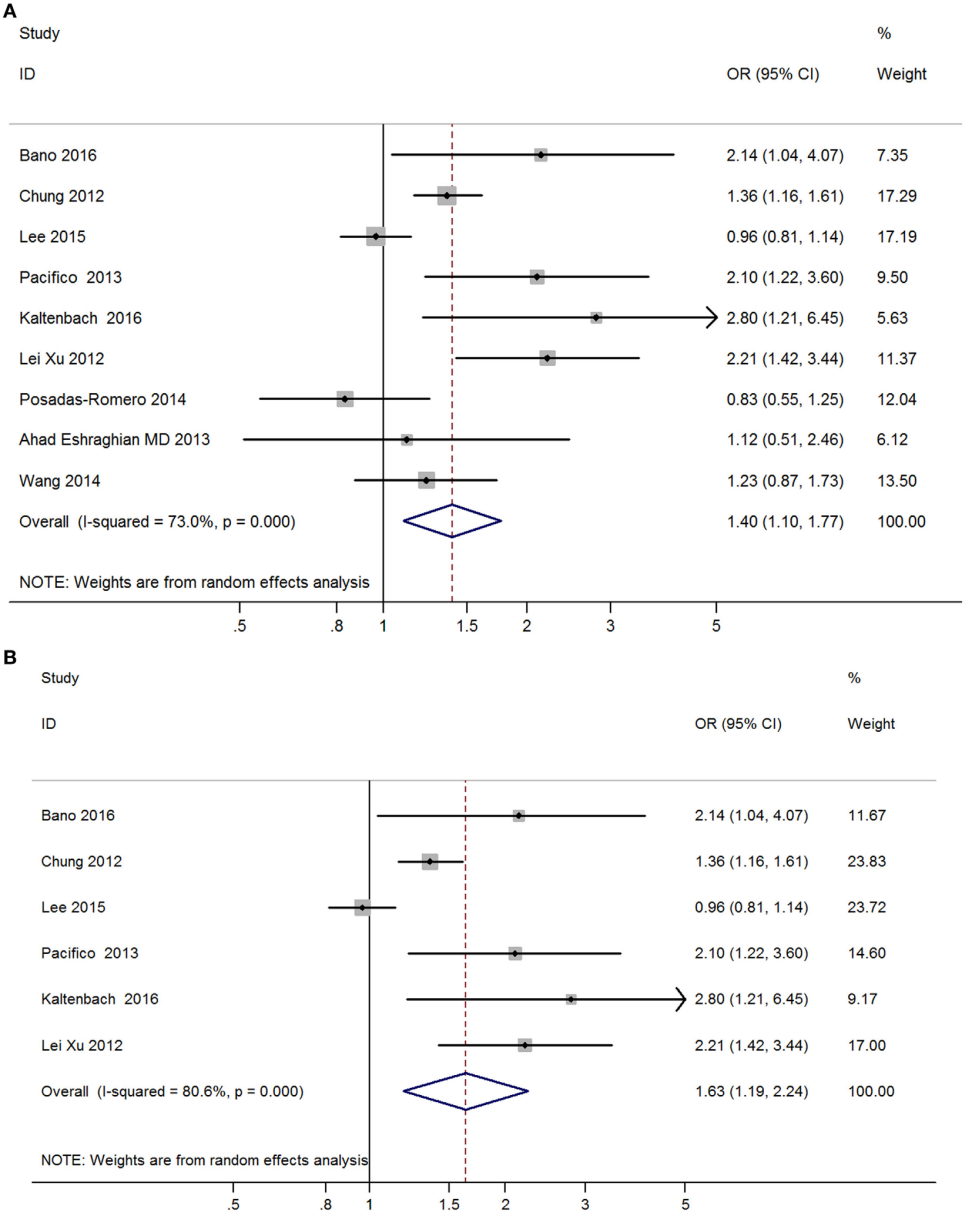
Forest plots in the meta-analysis of the relationship between subclinical hypothyroidism and non-alcoholic fatty liver disease (NAFLD). **(A)** Forest plot suggested that subclinical hypothyroidism was correlated with NAFLD. **(B)** Forest plot suggested that subclinical hypothyroidism was independently correlated with NAFLD.

Meta-analysis of nine studies found that subclinical hypothyroidism was significantly correlated with NAFLD (OR = 1.40, 95% CI 1.10–1.77, *P* < 0.006) (Figure 4A). After excluding three studies without providing adjusted ORs, meta-analysis of six left studies found that overt hypothyroidism was significantly correlated with NAFLD (OR = 1.63, 95% CI 1.19–2.24, *P* < 0.002) (Figure 4B).

**Figure 5:**
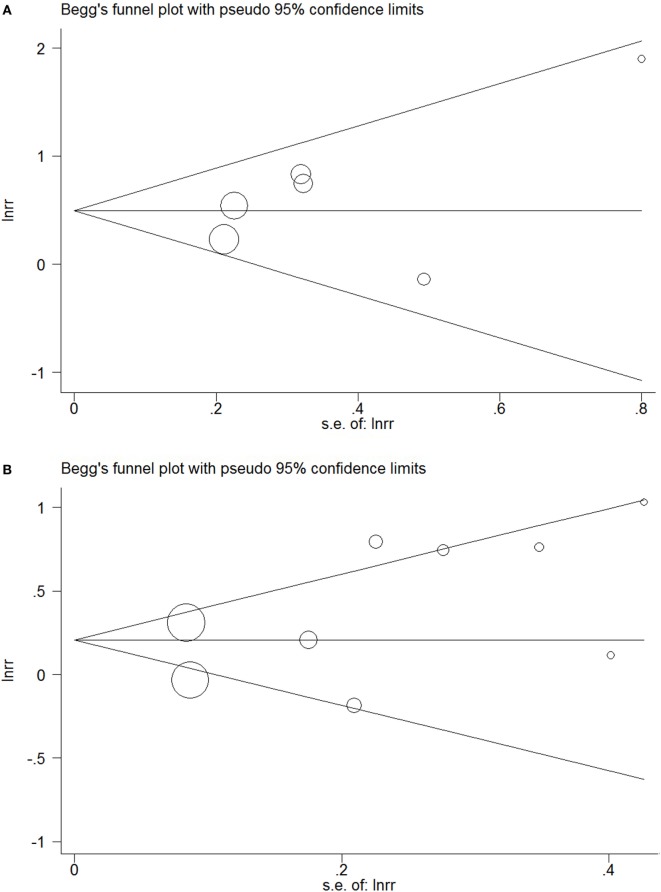
Funnel plots in the meta-analysis of the relationship between hypothyroidism and non-alcoholic fatty liver disease (NAFLD). **(A)** Funnel plot in the meta-analysis of the relationship between overt hypothyroidism and NAFLD. **(B)** Funnel plot in the meta-analysis of the relationship between subclinical hypothyroidism and NAFLD.

Funnel plots did not show obvious indications of publication bias (Figures 5A,B). The *P* values of Egger’s test in the meta-analyses relating overt hypothyroidism and subclinical hypothyroidism were 0.35 and 0.17, respectively.

## Discussion

Although previous studies propose that hypothyroidism might play a crucial role in the pathogenesis of NAFLD, some observational studies fail to find an obvious association between hypothyroidism and NAFLD. However, based on the results of the present meta-analysis, hypothyroidism plays an important role in the pathogenesis of NAFLD. The meta-analysis suggests epidemiological evidence for the obvious relationship between hypothyroidism and NAFLD, and the impact of hypothyroidism is independent from other known risk factors for NAFLD. Besides, both subclinical hypothyroidism and overt hypothyroidism are independently related to NAFLD. It is more remarkable that our study, which comprised 13 available studies from 11 countries, is the first meta-analysis integrating the evidence for the relationship between hypothyroidism and NAFLD.

According to our study, hypothyroidism independently increases the risk of NAFLD, which has some implications in the screening of hypothyroidism and NAFLD. It may be helpful for the screening of NAFLD among hypothyroidism patients since those patients are at higher risk of developing NAFLD. Meanwhile, it may also be helpful to identify hypothyroidism in patients with NAFLD and to give an appropriate treatment for hypothyroidism. Therefore, the results of this study is of great significance in the preventive medicine of hypothyroidism and NAFLD.

Our results demonstrate that either overt hypothyroidism or subclinical hypothyroidism independently increases the risk of NAFLD. Some studies have laid the foundation for the findings of the meta-analysis by providing some possible explanations for the molecular mechanism underlying the relationship between hypothyroidism and NAFLD. There are several possible mechanisms which can explain the relationship between hypothyroidism and NAFLD. First, obvious relations between hypothyroidism and metabolic changes have been reported, which include IR, dyslipidemia and obesity and they have important roles in the development of NAFLD (54, 55). Both IR and obesity are vital factors in the development of NAFLD, which are also common in hypothyroidism patients compared to those general population (56). IR can accelerate liver injury in NAFLD (57). Besides, Demir et al. found that hypothyroidism can cause NAFLD in rat models, and pointed out that obesity is one of the key factors in the relationship between hypothyroidism and NAFLD (29). The metabolic changes aforesaid among hypothyroidism patients can thus further result in the development of NAFLD (54, 55). Second, thyroid hormones can regulate lipid metabolism in the liver *via* thyroid hormone receptor β, and they can decrease cholesterol and triglyceride levels (58–60). It is worth mentioning that lower levels of thyroid hormones in hypothyroidism can increase the levels of cholesterol, low-density lipoproteins and triglyceride due to the delivery of hepatic fatty acids, but decrease the level of high-density lipoprotein (HDL), and thus can affect lipid metabolism (61). Therefore, patients with overt hypothyroidism often have fatty infiltration of the liver and thus have a higher risk for NAFLD (47). Hypercholesterolemia caused by hypothyroidism also plays an important role in the pathogenesis of NAFLD (41, 47). Thirdly, TSH itself can have a direct impact on the function of hepatocytes *via* TSH receptor signal (62–64). TSH can directly increase hepatic gluconeogenesis, repress hepatic bile acid synthesis, and cause hypercholesterolemia by decreasing HMG-CoA reductase phosphorylation (63–65), which further leads to the development of NAFLD. Finally, elevated oxidative stress markers can be observed in hypothyroidism patients (66). Oxidative stress is one of the mechanisms of NAFLD, and oxidative stress in liver tissue among hypothyroidism patients can cause cellular injury and IR *via* reducing beta-oxidation of fatty acids and increasing peroxidation of lipids (66).

Previous studies have found that thyroid hormones have important roles in regulating lipid metabolism in the liver *via* thyroid hormone receptors (58–60). Apart from thyroid hormones, thyroid hormone derivatives also have been reported to exert important effects on the lipid metabolism in the liver (67–69). 3,5-Diiodo-l-thyronine (T2) is the main type of thyroid hormone derivatives, and some studies has demonstrated that T2 has an lipid-lowering effect and can reduce the excess fat in cultured hepatocytes (68, 69). *In vivo* studies also have found that T2 has some important effects on the lipid metabolism of liver, such as reducing lipid accumulation and stimulating the pathways of lipid oxidation (70, 71). The above findings suggest that some thyroid hormone derivatives has protective effects against NAFLD, which can be promising treatments for NAFLD (67, 71).

According to the results of the present research, we found an obvious phenomenon that the correlation between overt hypothyroidism and NAFLD was more significant than that between subclinical hypothyroidism and NAFLD. As mentioned above, overt hypothyroidism is defined as having a much higher TSH level and lower T4 and T3 levels compared to subclinical hypothyroidism. The more significant correlation between overt hypothyroidism and NAFLD may be explained by the synergistic effects of higher TSH level and lower thyroid hormones in the pathogenesis of NAFLD, because TSH itself may induce hepatocyte steatosis *via* TSH receptor signal (72).

Although we carried out this meta-analysis very rigorously, several limitations should be acknowledged. First, in our meta-analysis, we included two cohort studies, seven cross-sectional studies, and four case–control studies. There was still lack of enough prospective cohort studies to evaluate the risk of NAFLD among hypothyroidism patients. Further prospective cohort studies are necessary to confirm our findings. Second, our research still had statistical heterogeneity, because there were inevitable differences in those included studies. For example, all the patients enrolled in those studies were from different countries. In the meta-regression analysis, we found that study design was a possible source of heterogeneity, but other sources were not identified. Third, most studies did not analyze the impact of thyroid hormone replacement therapy when exploring the risk of NAFLD among overt hypothyroidism or subclinical hypothyroidism patients. A recent study demonstrated that levothyroxine (LT4) had benefits on NAFLD in subclinical hypothyroid patients and LT4 supplementation played a beneficial role in delaying the development of NAFLD (23). More clinical trials are needed to evaluate the possible role of thyroid hormone replacement therapy in NAFLD among hypothyroidism patients.

In conclusion, our meta-analysis provides strong epidemiological evidence for the significant relationship between hypothyroidism and NAFLD. Both individuals with subclinical hypothyroidism and overt hypothyroidism are at a higher risk for the development of NAFLD than those with normal thyroid function. More prospective cohort studies are needed to further strengthen the relationship between NAFLD and hypothyroidism.

## Author Contributions

WH and J-aZ designed the study. WH, XA, LL, XS, QY, QL, and J-aZ contributed to the literature search, interpretation, writing, and proofreading of the manuscript. WH, XA, LL, and QY extracted data and performed data analyses.

## Conflict of Interest Statement

The authors declare that the research was conducted in the absence of any commercial or financial relationships that could be construed as a potential conflict of interest. The reviewer SP and handling Editor declared their shared affiliation.
